# Participatory intention and behavior towards riparian peri-urban forests management; an extended theory of planned behavior application

**DOI:** 10.3389/fpsyg.2024.1372354

**Published:** 2024-03-26

**Authors:** Rahim Maleknia, Jyran ChamCham

**Affiliations:** ^1^Forestry Department, Natural Resources Faculty, Lorestan University, Khorramabad, Iran; ^2^Agricultural Extention and Rural Development Department, Kermanshah, Iran

**Keywords:** pro-environmental behavior, peri-urban forest, participatory forest management, behavioral intention, mountaineers

## Abstract

**Introduction:**

Peri-urban forests play crucial role in quality of life and environment for citizens. To effectively utilize the services provided by these forests, it is essential to establish an integrated forest management system that aims to achieve a balance of all ecosystem services. This can be accomplished through a participatory approach that involves key citizen stakeholders. Mountaineers shape a specific group which have showed high pro-environmental behaviors to protect natural resources. This research aimed to examine the influencing factors on mountaineers’ intention to participate and their actual behavior in the management of riparian peri-urban forests in this field using extended theory of planned behavior.

**Methods:**

Environmental values and perceived barriers were added to original model as additional components to enhance its explanatory power. A sample size of 416 individuals was surveyed using a questionnaire. Data was analyzed using Smart-PLS.

**Results:**

The findings of the analysis revealed that the developed model accounted for 75.2% of the variance in mountaineers’ intention and 67.8% of behavior. The results demonstrated that three main components of model including attitudes, subjective norms and perceived behavioral control significantly influenced individuals’ intentions to participate in peri-urban forests management. Furthermore, intentions were cleared to have a positive influence on actual behavior in this context. Additionally, environmental values were found to be positively correlated with individuals’ intentions but not statistically significant behavior toward participate in urban forest management. Perceived barriers were found to have a negative impact on individuals’ intentions toward participate in urban forest management. The perceived barriers and behavior had not statistically significant relationship.

**Conclusion:**

The results of study provide valuable insights for the development of effective management strategies to promote mountaineers’ participation in riparian PUFs management. The study emphasizes the importance of environmental education and awareness campaigns targeted at mountaineers.

## 1 Introduction

Peri-urban along with urban forests play a crucial role in enhancing the quality of life, including health conditions and environmental quality, in urban environments (Conedera et al., 2015; Salbitano et al., 2016; Giannico et al., 2021; Reyes-Riveros et al., 2021). With the majority of the world’s population now residing in urban or peri-urban areas (World Bank, 2021), these forests have become an integral part of cities (Hoshyari et al., 2020). Riparian forests in peri-urban areas can be considered as specific type of PUFs, which offer various services to cities and citizens (Ordóñez et al., 2017; Ordóñez Barona et al., 2022; Peprah et al., 2022). These forests are particularly important due to their ability to connect terrestrial and aquatic ecosystems and establish links between urban areas and the natural environment (Gundersen et al., 2010). Riparian forests with special protection functions serve a crucial role in safeguarding forest ecosystems, with a particular emphasis on social, water, land, and soil protection (Tolkkinen et al., 2021; Chivulescu et al., 2023). It is essential to develop plans and strategies that focus on improving the management of these forests while considering the associated benefits (Galati et al., 2023; Shokrilahizadeh et al., 2023). The utilization of services provided by these forests necessitates the establishment of an integrated forest management system that strives for a balance of all ecosystem services through a participatory approach which involve important citizen stakeholders (Khazaei et al., 2018; Chivulescu et al., 2023). Public participation has received significant attention in environmental issues conservation (Tatari et al., 2019; Akbarizadeh et al., 2021; Sevianu et al., 2021; Savari et al., 2023b), as it can serve as a valuable tool for enhancing management practices. Therefore, it becomes crucial to engage urban communities comprehensively in the management of these natural resources in the urban and peri-urban areas. This involvement should encompass decision-making processes and implementation strategies to ensure successful management outcomes, citizen influence, and overall satisfaction (Sipilä and Tyrväinen, 2005). Therefore, evaluating and understanding the intentions and behaviors of citizens regarding their participation in peri-urban riparian forests is a crucial step in participatory management. Such evaluation can contribute to improving the effectiveness of strategies and plans aimed at enhancing PUFs management (Gulsrud et al., 2018; Galati et al., 2023).

The success of participatory management really depends on careful and thoughtful planning that fits well with the specific context and how much citizens are involved (Panyavaranant et al., 2023). Understanding the key factors is crucial for effective extension programs and public participation (Savari et al., 2023a). Evaluating and comprehensive understanding the factors that influence public participation, can help managers and policymakers tailor their approaches effectively to better align with the needs and preferences of the local community (Barabadi et al., 2020; Shokati Amghani et al., 2023). This can lead to more successful and sustainable management of urban and PUFs. A growing body of research have studied factors that influence the willingness of individuals to participate in urban forest management (Kirkpatrick et al., 2012; Zare et al., 2015; Dawes et al., 2018). These studies mainly focused on socio-economic factors, such as income level, education, gender, and occupation, which can significantly influence individuals’ willingness to engage in forest management. Additionally, Psychological factors, including attitudes, perceptions, and impact of society on person’s beliefs, also shape individuals’ intention to participate in urban forest management (Cebrián-Piqueras et al., 2020; Galati et al., 2023). Positive attitudes (Andrada, 2010; Ojeda-Revah et al., 2017; Tadesse and Teketay, 2017) towards nature and the environment, a sense of place attachment (Devine-Wright, 2009), and the belief in the importance of community involvement (López-Mosquera et al., 2014; Latifinia et al., 2022b) have been shown to can motivate individuals to actively participate in forest management initiatives. Personal attitudes toward participation, SNs, and individuals’ perceived control over their ability to participate are also influential in shaping individuals’ participatory intentions and behaviors in urban and PUFs. These factors serve as strong incentives for pro-environmental intentions and behaviors (Su et al., 2022).

As mentioned, previous studies have predominantly focused on the socio-economic factors influencing individuals’ willingness to participate in forest management or environmental activities. In the field of psychology, Behavioral willingness and behavioral intention are distinct concepts extensively studied to comprehend human behavior and decision-making. Although related, there are notable differences between these constructs (Simamora, 2022), Intentions reflect the motivational factors underlying actions and indicate the level of effort an individual plans to invest in a specific behavior (Ajzen, 1991). On the other hand, behavioral willingness signifies an individual’s openness to opportunities and their willingness to engage in a behavior in suitable situations (Pomery et al., 2009). In fact, willingness is more of a spontaneous social reaction rather than a carefully planned action (Gibbons et al., 1998). Researchers often assess willingness through self-reported measures, where individuals express their agreement or willingness to participate in a behavior or activity. Behavioral willingness is voluntary and can be influenced by situational factors, varying over time (Ajzen, 1991; Rivis and Sheeran, 2003). On the other hand, behavioral intention indicates an individual’s conscious decision to engage in a specific behavior in the future, reflecting their commitment and determination to act in a certain way (Ajzen, 2011). Intention is typically assessed using self-reported scales, where individuals indicate the extent to which they intend to carry out the behavior within a given timeframe. Behavioral intention is future-oriented and goal-directed, signifying a deliberate plan and indicating motivation and commitment to follow through with the action (Ajzen, 1991). Unlike willingness, intention tends to be relatively stable over time and is a robust predictor of actual behavior. Higher levels of intention are generally associated with a greater likelihood of engaging in the behavior (Sheeran and Webb, 2016). However, it is important to note that willingness does not always translate into intention, and intention does not guarantee actual behavior. Although, many studies explored willingness to participate in Peri- and urban forests (Moskell et al., 2010; Zare et al., 2015; Sin et al., 2022; Elton et al., 2023), there is a research gap about intention and behavior. Therefore, it is crucial to determine participatory intention and its translation into actual behavioral outcomes. Understanding the link between intention and behavior is essential for effective planning and implementation of participatory approaches in urban forest management. Mountaineers shape a specific group which have showed high pro-environmental behaviors to protect natural resources (Zarei et al., 2021; Sun et al., 2022). Urban and PUFs have been experienced high recreational demand during and after covid-19 pandemic (Samuelsson et al., 2020; Venter et al., 2021; Derks et al., 2023). The rising demand for recreational function of these forests not only poses increasing pressure on their ecosystems, but it also presents an opportunity to engage this specific segment of the community in the management of riparian PUFs. Therefore, this study investigates the intention and participatory behavior of mountaineers in the management of riparian PUFs, which is a novel research endeavor. By focusing on mountaineers and considering the riparian PUFs, this study aims to contribute to the enhancement of literature body in this field.

The main objective of this study is to examine the intention and participatory behavior of mountaineers in the management of riparian PUFs. Furthermore, this study aims to accomplish the following objectives:

1. Extendeb the basic model of theory of planned behavior to enhance the explanatory power of the model.
2. Identifying the psychological factors that influence the intention and behavior of mountaineers towards participate in riparian PUFs management.

## 2 Theorical framework of study

### 2.1 Theory of planned behavior

The TPB has been widely recognized as a powerful framework for understanding and predicting human behavior in various fields. Developed by Icek Ajzen, the TPB posits that behavioral intentions are influenced by three factors including attitudes, SNs, and perceived behavioral control (PBC) (Ajzen, 1991). By employing the TPB, we aim to gain insights into the factors that shape individuals’ intentions and their behavior to participate in PUF management0 activities. The following section elaborates on the three main components of the model, while also presenting the research hypotheses based on these components.

Attitudes refer to an individual’s positive or negative evaluations of participating in a particular behavior. This factor is first determinant of individuals’ intention to evaluate a behavior within a given context. Numerous studies have highlighted the significant impact of attitude on intention, demonstrating that individuals with a positive attitude are more inclined to engage in the behavior (Arvola et al., 2008; Empidi and Emang, 2021; Savari and Khaleghi, 2023). Emotional component, which encompasses individuals’ feelings about the subject, and the cognitive component, which focuses on their beliefs are two components of attitude (De Bruijn, 2010). Accordingly, we hypothesize that attitude has a positive correlation with intention.

*H1*: There is a positive correlation between individuals’ attitude and their intentions towards participating in peri-urban forest management.

Subjective norms reflect the perceived social pressure and expectations from significant others regarding the behavior. In fact, this component of theory captures the extent to which an individual’s intention to engage in a behavior is influenced by the approval or disapproval of significant others or social groups (Ajzen, 1991). People often base their behavior on their perception of others’ opinions, and intentions are strongly shaped by the influence of close relationships (Ajzen, 1991). SNs reflect the social pressure or influence that individuals experience when making behavioral choices (Sánchez et al., 2018; Lucarelli et al., 2020).

*H2*: There is a positive correlation between individuals’ SNs and their intentions towards participating in peri-urban forest management.

Perceived behavioral control refers to an individual’s perception of his/her ability to conduct the behavior successfully (Ajzen, 1991). Furthermore, in the TPB, the PBC can directly influence behavior in addition to the direct impact of intention on behavior (Koka et al., 2020; Hagger et al., 2022). Empirical studies have provided substantial evidence supporting these proposed effects (Pouta and Rekola, 2001; Karimi and Mohammadimehr, 2022; Latifinia et al., 2022a). Based on this theoretical framework, we hypothesize that PBC influence intention to participate in urban forest management and behavioral control and intention to engage in urban forest conservation and the PBC over such behavior will exert direct positive influences on participation in urban forest management.

*H3*: There is a positive correlation between individuals’ PBC and their intentions towards participating in peri-urban forest management.

*H4*: There is a positive correlation between individuals’ PBC and their behavior towards participating in peri-urban forest management.

In the TPB, intention to engage in the specific behavior is the most direct predictor of behavior (De Bruijn, 2010). Intention can be defined as conscious plans or decisions to performe or display a particular behavior which is influenced by attitude, SNs and PBC in turn affects on behavior (Ajzen, 1991). H5 of our study examines the correlation between individuals’ intention and behavior towards participate in urban forest management.

*H5*: There is a positive correlation between individuals’ intention and behavior towards participate in peri-urban forest management.

### 2.2 The extended theory of planned behavior

While the TPB has provided valuable insights into intention and behavior, researchers have extended the theory to incorporate additional factors and complexities to enhance its explanatory power and applicability. These extensions have been particularly relevant in the field of forest and environmental studies, where understanding human behavior is crucial for effective conservation and sustainable management. Various research examined the applicability of the extended TPB in predicting public support for forest conservation policies (Empidi and Emang, 2021; Huang et al., 2021; Negahdari et al., 2023; Savari and Khaleghi, 2023).

#### 2.2.1 Environmental values

Environmental values (EVs) is considered as the driving factor of moral norms which can influence decision-making regarding environmental protection (Li et al., 2022). This factor reflects individuals’ attitudes, beliefs, and concern for the environment. This component emphasizes the importance of intrinsic values, such as a sense of responsibility towards nature, appreciation for biodiversity, and the desire to protect and preserve natural resources within and has been applied in pro-environmental behavior studies (Han, 2015; Pradhananga et al., 2017; Fenitra et al., 2021). Strengthening individuals’ EVs can foster their intention and behavior to participate in urban forest management. Due to this potential, we considered this factor as a new component in the extended model and examined following hypothesizes:

*H6*: There is a possitive correlation between individuals’ EVs and their intentions towards participating in peri-urban forest management.

*H7*: There is a positive correlation between individuals’ EVs and their behavior towards participating in peri-urban forest management.

#### 2.2.2 Perceived barriers

Barriers can be defined as external or internal factors that prevent individuals from a specific intention and hinder the development of a behavior (Muttilainen and Vilko, 2022). Accordingly, perceived barriers (PBs) refer to the perceived obstacles or challenges that individuals perceive as hindrances to their engagement in specific behaviors. These barriers can encompass various factors, such as lack of time, lack of motivation, fear of injury, and a lack of support and resources can negatively influence intention and behavior (Rodrigues et al., 2023). This component has not been used broadly in forest issues, but has been applied in another fields including accepting environment friendly transportation (Chen et al., 2018), adoption of heat and flood adaptation Behaviors (Jacob et al., 2021) and adaptation of climate change among forest growers (Villamor et al., 2023). In forestry field, lack of reforestation knowledge, financial limitations, un-successful past experiences, and a lack of trust with conservation organizations are considered as barriers to participate in forest management (Powlen and Jones, 2019). Some research tried to uncover potential barriers for engaging public in participatory forest management (Zare et al., 2015; Khedrizadeh et al., 2017), but there is no or at least limited studies which considered PBs in extended TPB in forestry field. By recognizing and addressing these barriers, interventions and strategies can be designed to overcome them and enhance individuals’ motivation to participate. Secondly, the consideration of PBs as a component of the extended TPB recognizes the importance of contextual factors in shaping behavior. Moreover, by incorporating the component of PBs, the extended TPB acknowledges the direct influence of these barriers on behavior, highlighting their significance in understanding participation patterns. Therefore, we developed following hypothesizes for this component:

*H8*: There is a possitive correlation between individuals’ PBs and their intentions towards participating in peri-urban forest management.

*H9*: There is a positive correlation between individuals’ PBs and their behavior towards participating in peri-urban forest management.

**Figure 1:**
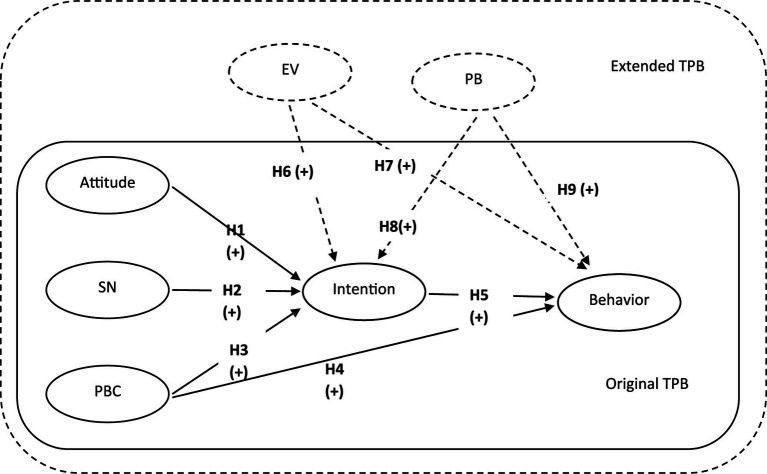
Theorical framework of study.

Figure 1 illustrate the original TPB and the extended version with two new components and hypothesizes of study.

## 3 Study area

This research was conducted on the hiking trails of Tehran. These trails, consisting of valleys and different terrains, have attracted a large population of mountaineers. Multiple rivers along the outskirts of Tehran’s hiking trails have led to the establishment of resort villages and numerous gardens. Additionally, the planting of numerous trees (Bozorgi et al., 2005) both artificially and naturally in the vicinity of these valleys has created a specific tree cover unique to the riverside forests. Darabad, Golabdareh, Darband, Velenjak, Darcheh, and Kan are among the major river valleys in Tehran, which, along with their vegetation cover, including gardens, meadows, and forests, serve as important environmental assets for water and soil conservation, as well as historical and cultural heritage of the city (Kaleji and Moslemi, 2015). These river valleys, with their tree cover, also have an impact on Tehran’s climate (Hamedani et al., 2021), and their preservation is essential for the well-being of the city.

## 4 Materials and methods

### 4.1 Sampling methods

The target population of this study comprised all mountaineers on climbing routes in the city of Tehran. A multistage stratified clustered sampling method was employed. Initially, all climbing routes in Tehran that featured riverside trees and forests were selected. Among them, four routes were chosen for the study. Then, equal numbers of samples were allocated to each route. The samples within each route were randomly assigned to individuals. Based on the guidelines provided by Krejcie and Morgan (1970), a sample size of 375 individuals was determined. However, to ensure greater accuracy a total of 416 questionnaires were distributed and completed. The purpose of the study and the contents of the questionnaire were explained to the participants in person, and the completed questionnaires were collected directly from them. Ample time was given to the participants to understand and complete the questionnaire. Participants were provided with a guarantee of confidentiality regarding their personal information and responses. They were assured that their identities would be kept anonymous and that their data would be treated with the utmost privacy and discretion. It was emphasized that the information they provided would only be used for the specific research study at hand and would not be shared with any external parties or used for any other purposes without their explicit consent. These measures were implemented to ensure the trust and willingness of participants to share their thoughts, opinions, and experiences openly and honestly, thereby contributing to the integrity and validity of the study.

### 4.2 Data collection

Data collection for this study took place during the spring and summer of 2023. A questionnaire was developed to gather the necessary data, consisting of two main sections. In section 1, a 5-point Likert scale was employed to measure various constructs, including attitude (3 items), SNs (3 items), PBC (3 items), intention (3 items), environment values (4 items), PBs (4 items), and behavior (5 items) (Table 1). These questionnaire items were derived from previous studies and can be found in Table 1, which provides a comprehensive overview of the constructs and scale items used. The second section of the questionnaire focused on collecting demographic information, such as age, gender, education level, income, and marital status.

**Table 1:** Constructs of model.

Construct	Observed variable	Measurement items	References
Attitude	AT1	I believe participating in conserving riparian pre-urban forests by mountaineers is wise	Sun et al. (2022) and Velardi et al. (2023)
	AT2	I believe participating in conserving riparian pre-urban forests by mountaineers is responsible.	
	AT3	Conserving riparian PUFs by mountaineers is necessary.	
Social Norms	SN1	If I participate in peri-urban trees conservation, my family, friends, and citizens approve my work.	Apipoonyanon et al. (2020) and Sun et al. (2022)
	SN2	Opinions of fellow mountaineers about participating in riparian pre-urban forests is important for me.	
	SN3	People who are important to me, except me to conserving riparian PUFs.	
Perceived Behavioral Control	PBC1	I have the basic knowledge and skills for participating in conserving riparian PUFs.	Savari et al. (2023a) and Sánchez et al. (2018)
	PBC2	I am confident that I can participate in conserve riparian PUFs.	
	PBC3	It is easy for me to participate in riparian PUFs conservation.	
Intention	Int1	I would like to participate in riparian PUFs conservation efforts during my mountain climbing, actively.	Galati et al. (2023) and Lucarelli et al. (2020)
	Int2	I have a strong intention to engage in riparian PUFs conservation efforts and incorporate sustainable practices in my climbing activities	
	Int3	I have intention to behave in conservative manner in regards to riparian PUFs during my mountain climbing.	
Environmental Value	EV1	I believe that conserving riparian PUFs in tracking routes contributes to the overall health and sustainability of the environment.	Fenitra et al. (2021) and Paletto et al. (2023)
	EV2	conserving the natural beauty and biodiversity of mountain climbing through riparian peri-urban conservation is personally important to me.	
	EV3	Valuing the intrinsic worth of peri-urban trees motivates me to actively participate in their conservation efforts	
	EV4	The riparian PUFs hold a special place for me, and I value their biodiversity, and contribution to ecological balance	
Perceived Barriers	PB1	The financial constraints and costs associated with participating in riparian PUFs, are perceived as barriers that influence active participate in these forests’ conservation initiatives	Buyinza et al. (2020), Chen et al. (2018), and Rodrigues et al. (2023)
	PB2	The concerns about personal safety within riparian PUFs, serve as barriers that affect engage in these forest conservation activities	
	PB3	The lack of management plans for riparian PUFs is perceived as a significant barrier to participate in conservation.	
	PB4	The perception of limited availability of information and knowledge about the benefits and procedures of participating in riparian PUFs acts as a barrier to participate in these forests’ conservation.	
Behavior	B1	I participate in the riparian PUFs conservation during climbing activities.	Ahmmadi et al. (2021) and Latifinia et al. (2022a)
	B2	I regularly participate in the planting and revitalization of riparian PUFs during my climbing	
	B3	I actively support and participate in the measures to support and conservation the riparian PUFs.	
	B4	During climbing, I warn people who destroy the forests.	
	B5	I inform the officials about the destruction in the forests.	

### 4.3 Reliability and validity of the questionnaire

To ensure the questionnaire’s quality, a team of experts with expertise in forestry, rural extension and education, environment, urban planning, and ecology evaluated its content and structure. In order to calculate Cronbach’s alpha coefficient, a pre-test was conducted using 30 questionnaires within a similar community. The data from the pre-test were used to calculate Cronbach’s alpha coefficient for all constructs included in the questionnaire ranging from 0.7 to 0.95. We also employed various indices to evaluate the robustness and validity of our questionnaire. Convergent validity was assessed using the Average Variance Extracted (AVE), which measures the extent to which a construct’s indicators collectively encapsulate its variance. A higher AVE is indicative of superior convergent validity. Furthermore, the questionnaire’s reliability was scrutinized through two metrics: Cronbach’s alpha coefficient and Composite Reliability (CR) (Dijkstra and Henseler, 2015). Cronbach’s alpha, a traditional gauge of internal consistency reliability, was employed to ascertain the degree of correlation among items within a scale. Concurrently, CR, a more contemporary and sometimes more accurate reliability measure than Cronbach’s alpha, was utilized to reinforce our evaluation of the questionnaire’s internal consistency. Discriminant validity testing was conducted to evaluate whether the measured constructs were distinct from each other and not overlapping. This testing is crucial in ensuring that each construct captures a unique underlying concept (Fornell and Larcker, 1981).

## 5 Results

### 5.1 Demographic characteristics of the study participants

Table 2 presents the sociodemographic characteristics of the study participants. Approximately 45% of the sample comprises female respondents, while males constitute 55%. In terms of marital status, the study includes both married individuals (49.5%) and unmarried individuals (50.5%). The age group ranging from 31 to 40 years old represents the largest proportion, with a total of 214 participants falling within this category. In relation to educational level, the majority of participants hold a bachelor’s degree (59%), followed by those with a high school diploma (18%), and individuals with a master’s degree or higher (12%). Among the income groups, individuals earning between 101 to 300 million Iranian rials per year show the highest frequency. Regarding occupation, participants engaged in permanent employment exhibit the highest prevalence, followed by individuals with part-time jobs (20.2%), housewives (8.4%), and unemployed respondents (8.4%). Students comprise 6.7% of the study participants.

**Table 2:** Demographic characteristics of the study participants.

Variables		Frequency	Percentage
Gender	Female	188	45.2
	Male	228	54.8
Marital Status	Single	210	50.5
	Married	206	49.5
Age	<20	21	0.05
	30–21	56	51.4
	40–31	214	21.6
	50–41	90	8
	>50	31	4
Educational Status	Illiterate	0	0
	Middle school	20	5
	Diploma	75	18
	Associate	25	6
	Bachelor	245	59
	Masters and higher	51	12
Income (million tomans)	<10	28	6.7
	11–100	144	34.6
	101–300	195	46.9
	301–500	21	5
	>500	28	6.8
occupation	House wife	35	8.4
	Full-time	234	56.3
	Part-time	84	20.2
	Unemployed	35	8.4
	student	28	6.7

### 5.2 Reliability and validity results

The reliability of the survey was ascertained through the computation of Cronbach’s alpha coefficients for all constructs, yielding values within the range of 0.852 to 0.932. This range indicates robust internal consistency, affirming a high level of reliability in the survey instrument. As delineated in Table 3, all constructs within the research model exhibited CR exceeding 0.792, along with Cronbach’s alpha coefficients surpassing 0.852. Moreover, the AVE values for all constructs in research model surpassed the threshold of 0.50 (Fornell and Larcker, 1981). This collective evidence underscores the satisfactory reliability and validity of all latent variables in the model. The result indicates that the items chosen to measure the constructs in the research were carefully selected, suggesting a thoughtful and deliberate approach. This outcome also suggests the potential for replicating the experiment, as the selected items have demonstrated their effectiveness in measuring the intended constructs.

**Table 3:** Reliability and validity test results.

Construct	Observed variable	CR	AVE	Cronbach’s alpha
Attitude	AT	0.957	0.880	0.932
Subjective Norms	SN	0.792	0.564	0.852
Perceived Behavioral Control	PBC	0.915	0.783	0.860
Intention	Int	0.956	0.878	0.930
Behavior	B	0.919	0.695	0.890
Environmental Value	EV	0.897	0.687	0.846
Perceived Barriers	PB	0.936	0.786	0.910

### 5.3 Discriminant validity

Discriminant validity testing was conducted to assess whether the constructs being measured are distinct and separate from each other. Discriminant validity testing helps confirm that the constructs of model are unique and not overlapping (Fornell and Larcker, 1981). All constructs demonstrated acceptable discriminant validity, indicating that they are distinct from each other and measure unique underlying concepts. This supports the validity of the measurement instrument used in assessing individuals’ attitudes, SNs, PBC, EVs, PBs, intentions, and behaviors. The results provide confidence in the theoretical framework of the model and support the use of these constructs in investigating relationships and effects within the model (see Table 4).

**Table 4:** The discriminant validity of the model constructs.

	Attitude	Behavior	Environmental value	Intention	Perceived barriers	Perceived behavioral control	Subjective norms
Attitude	0.938^a^						
Behavior	0.650^**^	0.834^a^					
Environmental value	0.646^**^	0.755^**^	0.823^a^				
Intention	0.696^**^	0.803^**^	0.820^**^	0.937^a^			
Perceived barriers	−0.636	−0.490^**^	−0.485^**^	−0.589^**^	0.886^a^		
Perceived behavioral control	0.725^**^	0.772^**^	0.750^**^	0.843^**^	−0.536^**^	0.885^a^	
Subjective norms	0.548^**^	0.710^**^	0.775*8	0.759^**^	−0.487^**^	0.638^**^	0.751^a^

a The square roots of AVE estimate.

^**^Correlation is significant at the < 0.01 level.

### 5.4 Structural model and hypothesis testing

**Figure 2:**
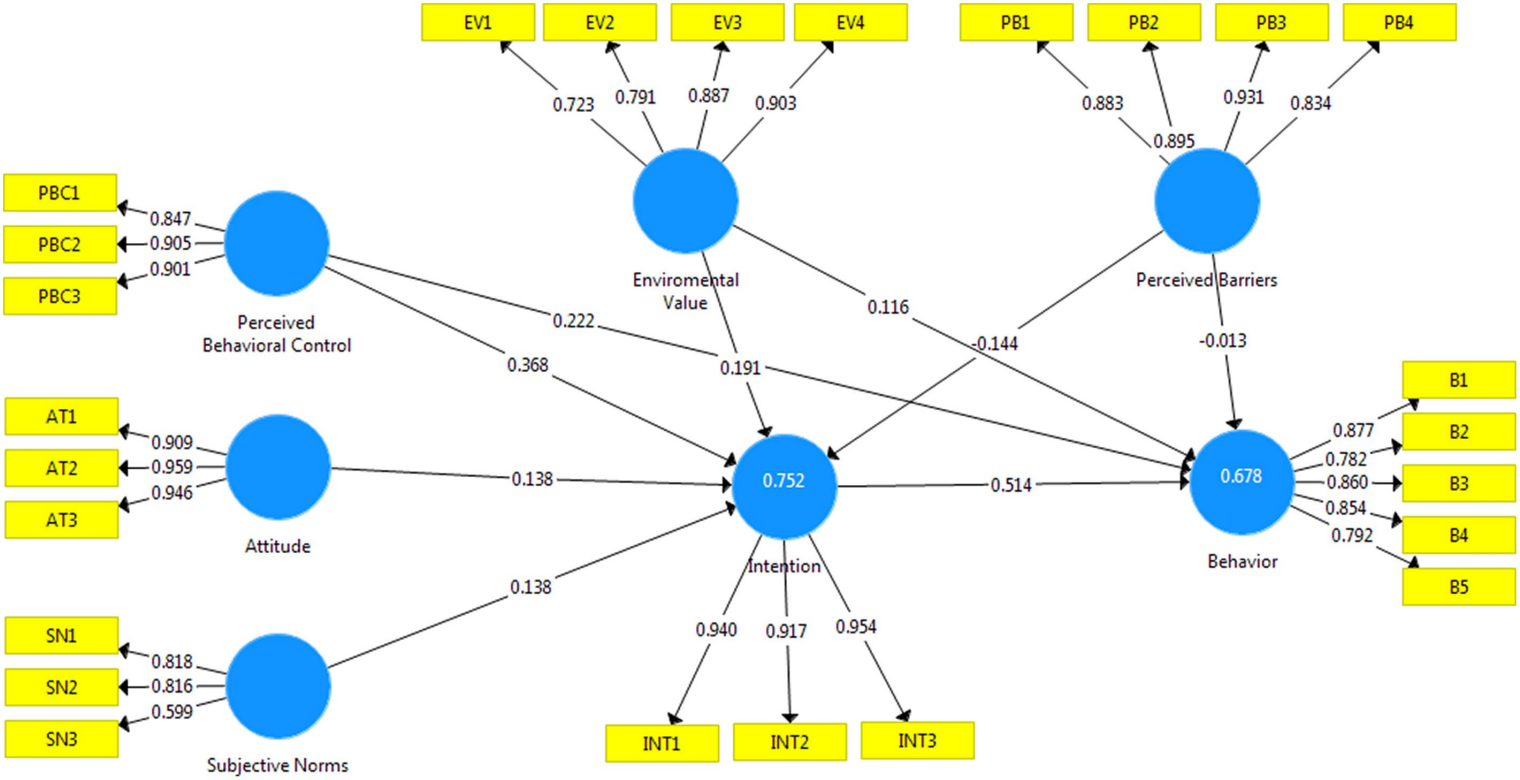
The extended model of research.

The results of the estimated structural model, depicted in Figure 2, reveal that the developed model accounts for 75.2% of the variance in mountaineers’ intention. Furthermore, the model explains 67.8% of individuals’ behavior. The results of the hypothesis test, presented in Table 5, provide insights into the direct and indirect effects as well. In the condition of the t value more than 1.96, the hypothesis is supported at 0.05 level significant (Hair et al., 2017). In line with the study’s proposed model, the results support several relationships among the basic constructs. Attitude (β = 0.138, t = 2.932, *p* < 0.01), SNs (β = 0.138, *t* = 2.947, *p* < 0.01), and PBC (β = 0.368, *t* = 4.619, *p* < 0.01) all positively influence individuals’ intentions, which, in turn, have a positive effect on their behavior. Additionally, PBC directly and positively impacts behavior (β = 0.222, *t* = 2.355, *p* < 0.01). Behavioral intention itself demonstrates a positive effect on behavior (β = 0.514, *t* = 12.941, *p* < 0.01). Furthermore, environmental value significantly influences intention (β = 0.191, *t* = 2.341, *p* < 0.01). However, the relationship between environmental value and behavior (H7) is not statistically significant (β = 0.116, *t* = 1.297, *p* > 0.01). Similarly, PBs show a significant negative relationship with intention (β = −0.144, *t* = 3.690, *p* < 0.01), but the relationship between PBs and behavior (H9) is negative and not statistically significant (β = −0.013, *t* = 0.444, *p* > 0.01). Based on these findings, hypotheses H1, H2, H3, H4, H5, H6, and H8 are supported, indicating the significant influence of attitudes, SNs, PBC, intention, and environmental value on behavioral outcomes. However, the relationships between environmental value and behavior (H7), as well as PBs and behavior (H9), are not statistically significant.

**Table 5:** Results hypotheses test.

Hypotheses	Path	Std. Beta	Std. Error	*t*-value	*p*-value	f2	Q2	Result
H1	AT→INT	0.138	0.047	2.932	0.004	0.19	0.655	Supported
H2	SN→INT	0.138	0.047	2.947	0.003	0.21	0.184	Supported
H3	PBC→INT	0.368	0.080	4.619	0.001	0.32	0.510	Supported
H4	PBC→B	0.222	0.094	2.355	0.019	0.21	0.510	Supported
H5	INT→B	0.514	0.040	12.941	0.001	0.34	0.653	Supported
H6	EV→INT	0.191	0.082	2.341	0.020	0.25	0.465	Supported
H7	EV→B	0.116	0.090	1.297	0.195	0.029	0.465	Reject
H8	PB→INT	−0.144	0.036	3.960	0.001	0.41	0.594	Supported
H9	PB→B	−0.013	0.029	0.440	0.660	0.075	0.594	Reject

## 6 Discussion

The present study aimed to examine the influencing factors on mountaineers’ intention to participate in the management of riparian PUFs. The results of the structural model analysis revealed that the developed model accounted for a substantial proportion of the variance in mountaineers’ intention (75.2%) and behavior (67.8%). These findings contribute to our understanding of the factors that drive mountaineers’ intentions and behaviors regarding riparian PUFs management. Consistent with previous research (Lam, 1999; Butt et al., 2021), our study found that attitudes, SNs, and PBC significantly influenced participants’ intentions to participate in riparian PUFs management, thus hypotheses H1, H2, H3, H4 and H5 are supported. Studies also showed that these constructs can affect pro-environmental behavior of mountaineers (Sun et al., 2022; Wang and Wang, 2022; Mohammadi et al., 2024). Research also showed that these factors can result in pro-environmental behavior among visitors of urban forests (López-Mosquera et al., 2014; Huang et al., 2021). This suggests that mountaineers who hold positive attitudes towards riparian PUFs management, perceive social pressure from their peers or relevant groups to participate, and feel a sense of control over their ability to engage in forest management activities are more likely to express intention to participate. These results show the importance of these constructs in predicting behavioral intentions and subsequent behaviors of individuals (Rivis and Sheeran, 2003; Ajzen, 2011). The positive relationship between attitude and intention confirms that individuals’ positive evaluations or beliefs toward performing a behavior are likely to increase their intention to engage in that behavior (Ajzen, 1991). Similarly, SNs were found to positively influence intention. This suggests that mountaineers’ perceptions of social pressures and expectations inform their intentions to engage in a particular behavior. The influence of SNs on intentions has been widely supported in the literature (Armitage and Conner, 2010; Ahmmadi et al., 2021; Savari and Khaleghi, 2023). PBC was also found to significantly impact intention, indicating that individuals’ beliefs about their ability to perform the behavior have a direct influence on their intentions. This finding is consistent with the concept of self-efficacy, which suggests that individuals’ confidence in their ability to execute a behavior is a crucial factor in determining their intentions and subsequent actions (Ajzen, 1991). Mountaineers, as a distinctive group within society, are individuals who share a deep passion and connection with mountains (Wang and Wang, 2022). Given their affinity for these natural environments, mountaineers often possess the necessary skills and knowledge related to the management of riparian PUFs. Consequently, the constructs of SN and PBC play a significant role in shaping their intention to engage in pro-environmental behaviors, specifically in the context of forest management. This highlights the immense potential that mountaineers hold in contributing to the management of these forests, and it is crucial for forest managers to recognize and harness this potential. It is important to note that these findings provide valuable insights into hikers’ intentions specifically related to riparian PUFs conservation. The unique characteristics of these forest areas, such as their proximity to urban environments and ecological significance, may have influenced the relationships observed in this study. Future research should aim to replicate and expand upon these findings in different geographical contexts and with diverse participant samples. Furthermore, our study identified a significant positive relationship between behavioral intention and actual behavior, supporting the notion that intention serves as a reliable predictor of subsequent behavior. This finding aligns with previous studies on environmental conservation behaviors (Zhong et al., 2019; Jiang et al., 2021; Baghernejad et al., 2023) and underscores the relevance of understanding and targeting individuals’ intentions to promote their engagement in forest management activities.

In terms of the specific factors examined in this study, the influence of environmental value on behavioral outcomes was found to be significant in shaping participants’ intentions to participate in riparian PUFs management. This finding aligns with previous studies that have highlighted the role of knowledge about EVs in motivating pro-environmental behaviors (Seeland et al., 2002; Kaiser and Scheuthle, 2003; Fenitra et al., 2021). Studies have found that environmental value positively affects individuals’ intentions to engage in pro-environmental behaviors (Kaiser et al., 1999; Li et al., 2022). Interestingly, the relationship between environmental value and behavior was not significant in this study. This suggests that while individuals’ values and beliefs about the environment may influence their intentions, they may not directly translate into observable behaviors. This discrepancy could be attributed to various factors, including the complexity of translating intentions into concrete actions and the presence of other contextual or situational factors that may affect behavior in the specific context of riparian PUFs management. It can be discussed that people with knowledge about EVs of PUFs have positive behavioral intention (Jama et al., 2023), but translation of this intention into behavior needs some measures including training and education. Furthermore, EVs may be more abstract and general in nature, focusing on the overall well-being of the environment and its preservation (Stern et al., 1999). However, behaviors are often context-specific and require concrete actions within a particular setting or situation. The specificity and practicality of pro-environmental actions may not always align with individuals’ abstract EVs, leading to a disconnect between intentions and behaviors.

Furthermore, we added PBs as a new component to the initial model. This component had a significant negative impact on individuals’ intentions to participate in the management of riparian PUFs, but it had a non-significant negative effect on their behavior. The results suggest that mountaineers who perceive fewer obstacles or difficulties are more likely to express intention to participate in forest management. This component has not been explored in field of forestry. But, the negative correlations between PBs and intention and actual environmental behavior (Chen et al., 2018; Jacob et al., 2021), physical activity (Rodrigues et al., 2010) were observed. Some barriers including lack of time, lack of trust in participatory management plans, financial limitations, and un-successful past experiences, are considered as barriers to participate in forest management (Zare et al., 2015; Khedrizadeh et al., 2017; Powlen and Jones, 2019). In fact, participatory behaviors often require sustained effort, time, and resources, which can pose practical challenges for individuals. This discrepancy between intention and behavior in term of barriers highlights the importance of considering factors which can either facilitate or hinder the translation of intentions into actual behavior. However, translating intentions into behavior is a more complex process. Individuals may have positive attitude, PBC and EVs which shape their intention to participate in PUFs management, but face some barriers such as time constraints or logistical challenges, situational constraints, lack of resources, and competing priorities that prevent them from actively participating in conservation activities, regardless of their intentions.

## 7 Conclusion

This research aimed to investigate mountaineers’ intention and actual behavior to participate in riparian PUFs management using the TPB. Through the analysis of data collected from a sample of 395 mountaineers in study area, several key findings emerged. The results demonstrated that attitudes, SNs, and PBC significantly influenced individuals’ intentions to participate in PUFs management. Furthermore, intentions were found to have a positive impact on actual behavior in this context. These findings align with the hypothesized relationships and support the validity of the TPB in explaining mountaineers’ engagement in riparian PUFs. Additionally, EVs were found to be positively correlated with individuals’ intentions to participate in urban forest management. However, the relationship between EVs and actual behavior was not statistically significant. This suggests that while individuals may hold positive EVs, other factors may influence their actual engagement in forest management activities. Furthermore, PBs were found to have a negative impact on individuals’ intentions to participate in urban forest management. However, the relationship between PBs and behavior was not statistically significant. This implies that although PBs may hinder individuals’ intentions, their actual behavior might not be influenced by PBs.

The incorporation of PBs and EVs into the basic model has improved its explanatory power. To foster mountaineers’ engagement, it is essential to address these variables effectively. Efforts aimed at addressing barriers to participation in PUFs management and strengthening mountaineers’ understanding of the environmental values associated with these forests are crucial for fostering active engagement. It is necessary to undertake measures that effectively mitigate the obstacles hindering participation and enhance knowledge about the significance of environmental conservation in PUFs management. By implementing these strategies, managers can effectively overcome the barriers related to perception and motivate mountaineers to actively contribute to the sustainable management of riparian PUFs. This collective effort will benefit both present and future generations, ensuring the preservation of riparian PUFs ecosystems.

This study is subject to several limitations. Firstly, the quantitative nature of the research focuses primarily on establishing causal relationships between variables, potentially overlooking the nuanced understanding of mountaineers’ intentions and behaviors towards participation in riparian PUFs management. Combining qualitative research methods with quantitative analysis would provide a more comprehensive understanding of the influencing factors. Secondly, reliance on self-reported data introduces potential biases and social desirability effects, which may lead to overestimation or underestimation of true intentions. Future studies should consider incorporating objective measures or behavioral observation to complement self-report data, providing a more accurate reflection of participants’ engagement in riparian PUFs management. Furthermore, the generalizability of the findings may be limited due to the specific context and sample characteristics of the study. Factors influencing mountaineers’ intentions and behaviors to participate in PUFs management for can vary across different geographical areas, cultural backgrounds, and socio-economic contexts. Caution should therefore be exercised when extrapolating the results to other populations or regions. Future research should aim to include diverse samples and multiple contexts to enhance the external validity of the findings.

## 8 Theoretical and practical implications

This study contributes to the literature by applying the TPB to the context of mountaineers’ participation in riparian PUFs management. The findings confirm the applicability of TPB in understanding the intentions and behaviors of mountaineers in this specific domain. The study identifies several factors that significantly influence mountaineers’ intention to participate in urban forest management. Attitudes, SNs, and PBC all play crucial roles in shaping individuals’ intentions. These findings provide empirical evidence supporting the importance of these factors within the TPB framework. The study underscores the positive correlation between individuals’ intentions and their actual behavior in the context of participating in urban forest management. This finding aligns with previous research and emphasizes the predictive power of intentions in driving behavior. The study reveals the significant influence of EVs on individuals’ intentions to participate in forest management. This finding highlights the importance of fostering EVs among mountaineers and suggests that promoting environmental awareness may enhance their intention to engage in conservation activities.

The findings of this study provide valuable insights for the development of effective management strategies to promote mountaineers’ participation in riparian PUFs management. By targeting attitudes, SNs, and PBC, managers can design interventions that encourage positive intentions and subsequent behaviors. The study emphasizes the importance of environmental education and awareness campaigns targeted at mountaineers. By promoting EVs and highlighting the benefits of conservation efforts, such initiatives can enhance mountaineers’ intentions to participate in urban forest management. Although the study did not find a significant relationship between PBs and behavior, addressing PBs remains important in facilitating participation. Managers should identify and address the specific obstacles that mountaineers perceive, such as lack of time or resources, to encourage greater engagement. Given the influence of SNs on mountaineers’ intentions, involving key stakeholders, such as fellow climbers, outdoor organizations, and community members, can foster a supportive social environment that promotes participation in forest management. Collaborative efforts and partnerships can strengthen the social norms surrounding conservation behaviors. The study’s findings can inform policy development related to riparian PUFs management. By recognizing the factors influencing mountaineers’ intentions and behaviors, policymakers can design targeted policies and incentives to encourage greater participation and sustainable forest management practices.

## 9 Recommendation for future study

Further investigation of potential mediating and moderating factors could enhance the understanding of the relationships identified in this study. For instance, exploring the role of environmental knowledge, personal values, or social support as mediators or moderators may provide a more nuanced understanding of the mechanisms underlying mountaineers’ intentions and behaviors in forest management. Investigating the influence of contextual factors on mountaineers’ intentions and behaviors could be valuable. Factors such as the accessibility of forest areas, the presence of environmental policies, or the availability of educational resources may impact mountaineers’ engagement in forest management. Exploring the interactions between individual-level factors and contextual factors could provide a more comprehensive understanding of the drivers of participation. This study focused on immediate intentions and behaviors, but long-term behavior change is crucial for sustainable forest management. Future research could examine the factors that facilitate long-term engagement and the maintenance of environmentally responsible behaviors among mountaineers. Understanding the determinants of sustained participation over time would contribute to more effective conservation strategies. Conducting comparative studies across different outdoor recreational activities or geographical locations could provide valuable insights into the specific influences and dynamics of mountaineers’ participation in forest management. Comparing the attitudes, intentions, and behaviors of mountaineers with other outdoor enthusiasts or different cultural contexts could highlight unique factors and inform tailored interventions. Involving multiple stakeholder groups, including local communities, forest managers, and policymakers, in future research could provide a holistic understanding of the challenges and opportunities for mountaineers’ participation in forest management. Examining the perspectives and interactions among different stakeholders would facilitate the development of comprehensive strategies and policies.

## Data availability statement

The raw data supporting the conclusions of this article will be made available by the authors, without undue reservation.

## Ethics statement

Ethical review and approval was not required for the study on human participants in accordance with the local legislation and institutional requirements. Written informed consent was not required to participate in this study in accordance with the local legislation and institutional requirements.

## Author contributions

RM: Conceptualization, Data curation, Methodology, Project administration, Supervision, Writing – original draft, Writing – review & editing. JC: Data curation, Investigation, Methodology, Software, Writing – review & editing.
